# Supplementary material for: Splice-Junction-Based Mapping of Alternative Isoforms in the Human Proteome
Source: Cell Rep. Author manuscript; Available in PMC 2020 Jan 15. (PMC6961840; doi:10.1016/j.celrep.2019.11.026)
Supplement: 3 [file NIHMS1546469-supplement-3.zip › DF2/PXD0010154/Esophagus-205-P24844-DGFIDKEDLHDMLASLGF.pdf]

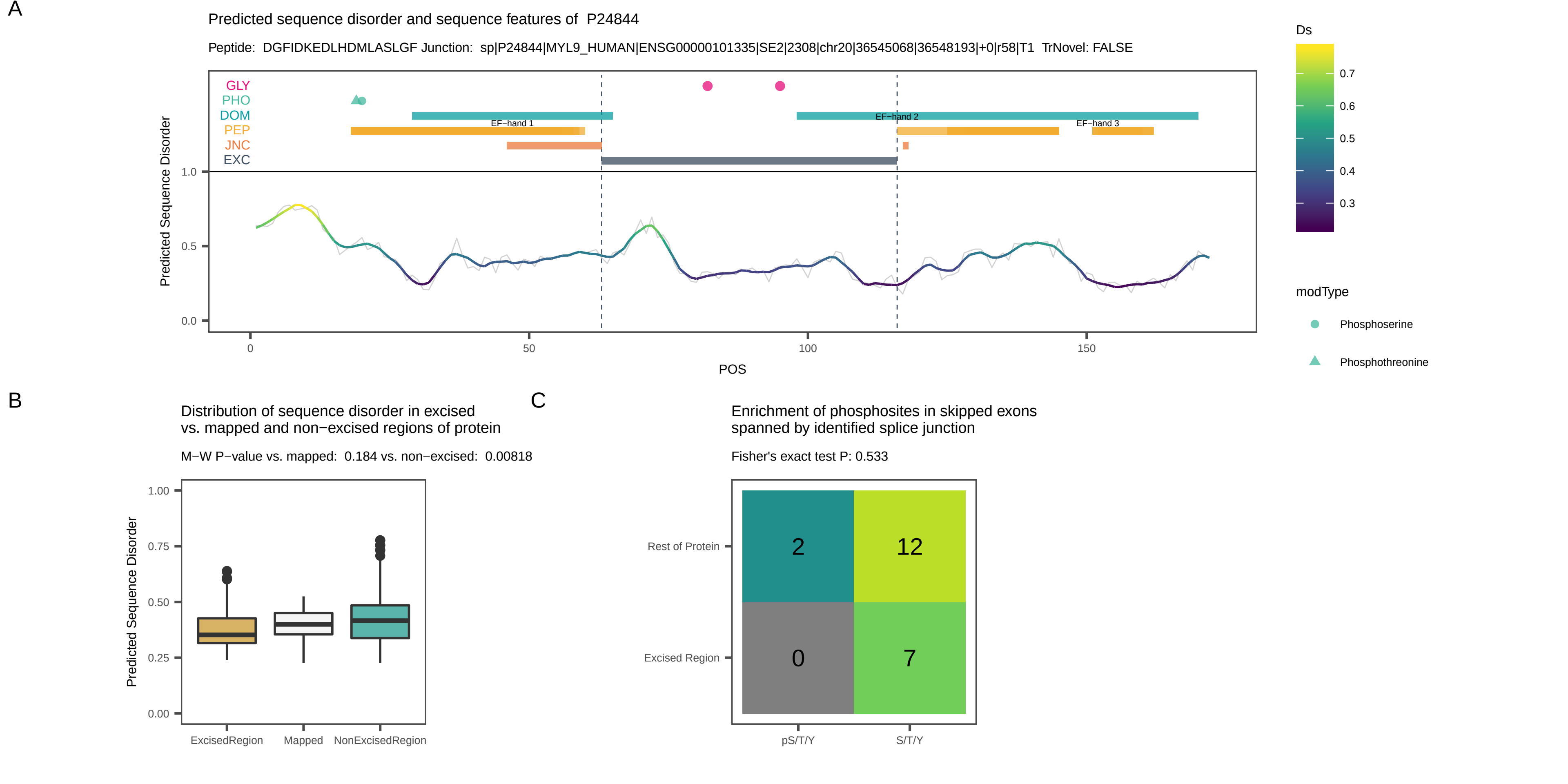
A
Predicted sequence disorder and sequence features of P24844
Peptide: DGFIDKEDLHDMLASLGF Junction: sp|P24844|MYL9_HUMAN|ENSG00000101335|SE2|2308|chr20|36545068|36548193|+0|r58|T1 TrNovel: FALSE
GLY
PHO
DOM
PEP
JNC
EXC
EF−hand 1
EF−hand 2
EF−hand 3
Predicted Sequence Disorder
1.0
0.5
0.0
0
50
100
150
POS
Ds
0.7
0.6
0.5
0.4
0.3
modType
Phosphoserine
Phosphothreonine
B
Distribution of sequence disorder in excised vs. mapped and non−excised regions of protein
M−W P−value vs. mapped: 0.184 vs. non−excised: 0.00818
Predicted Sequence Disorder
1.00
0.75
0.50
0.25
0.00
ExcisedRegion
Mapped
NonExcisedRegion
C
Enrichment of phosphosites in skipped exons spanned by identified splice junction
Fisher's exact test P: 0.533
Rest of Protein
Excised Region
2
12
0
7
pS/T/Y
S/T/Y
